# Patients experiences with multiple sclerosis disease-modifying therapies in daily life – a qualitative interview study

**DOI:** 10.1186/s12913-021-07012-z

**Published:** 2021-10-22

**Authors:** Anna Sippel, Karin Riemann-Lorenz, Jutta Scheiderbauer, Ingo Kleiter, Rebecca Morrison, Christopher Kofahl, Christoph Heesen

**Affiliations:** 1 Institute of Neuroimmunology and Multiple Sclerosis (INIMS), University Medical Center Hamburg-Eppendorf (UKE), Martinistrasse 52, 20246 Hamburg, Germany; 2 Patient representative, Trier, Hamburg, Germany; 3 Marianne-Strauß-Klinik, Behandlungszentrum Kempfenhausen für Multiple Sklerose Kranke gGmbH, Berg, Germany; 4 Independent researcher, Berlin, Germany; 5 Institute of Medical Sociology, University Medical Center Hamburg-Eppendorf (UKE), Hamburg, Germany; 6 Department of Neurology, University Medical Center Hamburg-Eppendorf (UKE), Hamburg, Germany

**Keywords:** Multiple sclerosis, Decision making, Web-based experiential information, Patient experiences, Thematic analysis, Qualitative study

## Abstract

**Background:**

Besides coping with a disease with many uncertainties, people with relapsing-remitting multiple sclerosis face complex decisions concerning disease-modifying therapies (DMTs). In an interview study, we aimed to assess patients’ experiences with DMTs.

**Methods:**

Problem-centred interviews were conducted with 50 people with relapsing-remitting multiple sclerosis in Germany using maximum variation sampling and covering all licensed DMTs. Data were analysed thematically using deductive and inductive categories.

**Results:**

47 of 50 patients had treatment with at least one of the approved DMTs. The main themes were: (1) starting a DMT, (2) switching to another DMT, (3) discontinuing a DMT, and (4) multiple sclerosis without starting a DMT. Different intercorrelated factors influenced the decision-making processes for or against a DMT. Individual experiences with DMTs in daily life contained the effort in administration, success, and failure of DMTs, coping strategies and well-being without DMTs. The decision-making process for or against a DMT and the use of those treatments can be understood as a constant, continually shifting process, complicated by different factors, which change over time. Experiences with DMTs were characterized by attempts to handle uncertainty and to (re)gain control and integrate adaptivity into one’s life.

**Conclusions:**

The study provides a rich and nuanced amount of patients’ experiences with DMTs. The findings demonstrate the importance for practitioners to look at current life circumstances of patients with multiple sclerosis when recommending a DMT and to promote and enable patients to make informed decisions.

**Supplementary Information:**

The online version contains supplementary material available at 10.1186/s12913-021-07012-z.

## Background

Multiple Sclerosis (MS) is a chronic, inflammatory, and degenerative disease of the central nervous system. In Germany, about 224.000 people with MS (pwMS) live with the disease, mostly diagnosed at age 20 to 40. Three times as many women as men are affected [1]. Three major forms of disease course are distinguished: (1) Relapsing-remitting MS (RRMS) is the most frequent type, which is characterized by unpredictable relapses followed by periods with no new signs of disease activity. (2) Nearly 15 years after diagnosis, about half of the patients with RRMS develop secondary progressive MS (SPMS) and (3) about 10–15 % are diagnosed with primary progressive MS (PPMS) [2].

In the last decade, a wide range of disease-modifying therapies (DMTs) for RRMS became available and continues to emerge. DMTs aim at reducing the number and severity of inflammatory attacks and delaying the onset of the progressive phase of MS. All of them can have mild to severe adverse effects. Without data on the long-term effectiveness, the true impact on patients remains unclear [3]. Several factors can affect the choice for or against a specific DMT: disease activity, disability status, health-care delivery, previous treatments, risk tolerance, potential adverse effects, patients’ preferences, information provision, and the physician’s experience. Besides, patients use other approaches such as lifestyle interventions, rehabilitation, and complementary alternative therapies [4]. Choosing the appropriate therapy involves taking multiple factors into account, which constantly challenges the patient and the physician [5–8].

When exploring patients’ experiences with DMTs, previous studies have focused on influencing factors in the decision-making for a DMT, on the use of DMTs [6, 9], and decision-making preferences (patient-centred, shared and physician-centred) [5, 10, 11]. However, a greater insight into patients’ individual experiences using DMTs in daily life, for switching and discontinuing DMTs and for deciding not to start treatment with a DMT is under-researched. We addressed this gap with a qualitative study on experiences of RRMS patients with DMTs from the decision to choose a DMT (or not) to discontinuing a DMT to give valuable information and directions for the further decision-making process on DMTs in treatment and counselling.

## Methods

### Study design

This qualitative interview study is part of the project “Patient Experiences with Multiple Sclerosis”, aiming to evaluate in a study whether patients’ experiences may help other patients with RRMS in their DMT related decision-making processes as a supplement to evidence-based information. Based on this qualitative interview study, we are developing a website providing videos, audio recordings, and written excerpts of patients’ experiences with MS.

### Recruitment and participants

A maximum variation sampling strategy [12] was used to cover heterogeneous patients’ experiences with all DMTs licensed in Germany trying to balance positive and negative experience in relation to the perceived effect and handling in every-day life. We intended to gather experiences from at least 3 patients for each DMT. At the time of the interview study (March 2018 - May 2020), 11 pharmaceutical substances (Glatiramer acetate, Interferon-beta, Teriflunomide, Dimethyl fumarate, Alemtuzumab, Natalizumab, Ocrelizumab, Cladribine, Fingolimod, Mitoxantrone, Daclizumab) were approved for RRMS in Germany. Also, we sought out pwMS who had not started any DMT. The aim was to interview 40–50 pwMS. PwMS were recruited from clinics, rehabilitation centres, and patient associations located in Germany. PwMS were eligible if they were at least 18 years old and had a RRMS diagnosis. Individuals were excluded from the study if they had PPMS, had severe cognitive impairment, and/or did not speak German. After the first 30 interviews, a constant check for maximum variation was made for further recruitment.

### Data collection

A problem-centred interview guide [13] was mutually developed with the advisory board consisting of representatives of pwMS, researchers, neurologists, and the expert panel on qualitative methods at our clinic. The interview guide contained open-ended and closed questions on experiences with diagnosis, MS in everyday life, and with disease management approaches (DMTs, lifestyle intervention, alternative medicine, rehabilitation) (see Additional file 1). The interview guide was pre-tested with five pwMS to ensure clarity and appropriateness of length. As no further adjustment seemed necessary, these interviews were fully integrated in our study. Additionally, demographic data was collected as well as MS-related information, e.g. the ‘Patient Determined Disease Steps’ (PDDS), which asks for the patient-reported disability (from 0 = normal to 8 = bedridden) [14]. All interviews were conducted by a research associate experienced in qualitative research, but without medical education in MS and without MS diagnosis. The interviews were audio- and/or videotaped and transcribed. They were conducted at the homes of pwMS, at hotels, in clinics and a rehabilitation centre, and one at a workplace. This approach allowed people with mobility restrictions to take part in the interview study. Furthermore, the preferred location can make pwMS feel comfortable when sharing personal stories and experiences [15]. The interviewees received an incentive of 20 €.

### Data analysis

All interview transcripts were analysed thematically following the six phases of Braun and Clarke [16] using a mix of deductive and inductive analytic approaches. After familiarisation with the data and creation of initial codes, identified themes were predominantly data-driven. Codes were structured by potential major themes and sub-themes and reviewed. Afterwards, an ongoing reflection and refinement on the themes followed (a) by peer discussion (b) by consideration of the current state of research, and (c) during the writing process. The analysis was conducted computer-assisted via the program MAXQDA Analytics Pro 2018.

## Results

**Fig. 1:**
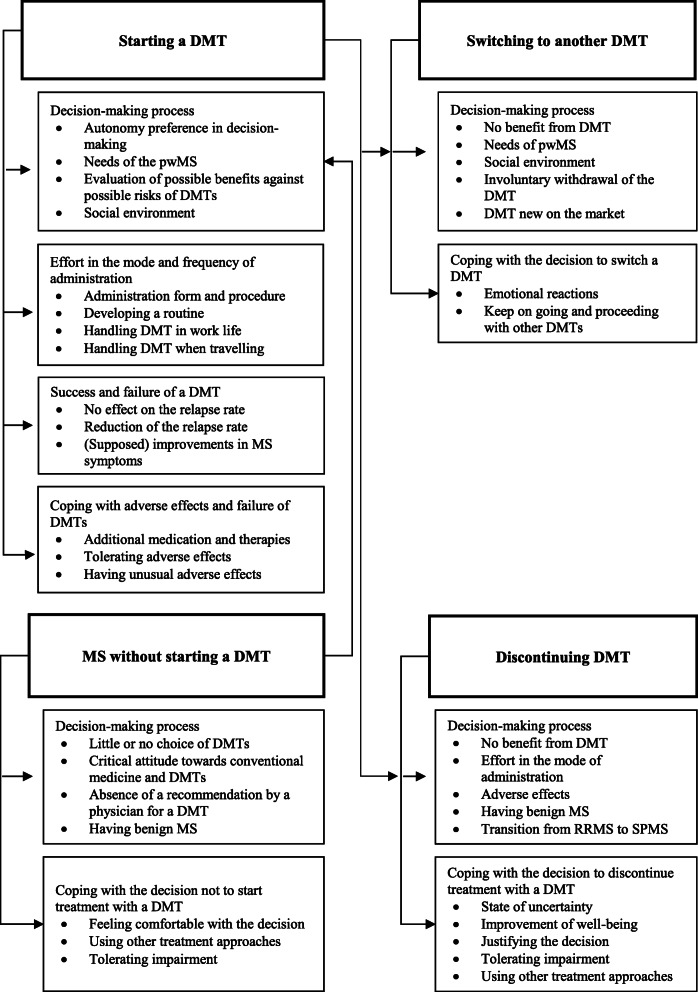
Thematic map of themes and sub-themes of pwMS’ experiences with DMTs

In this study, 50 pwMS were interviewed. Interviews lasted between 20 and 97 min (mean = 45.6). 22 interviews took place in Northern Germany, 23 in the South, and 5 in the East. Most people already had experience with more than two DMTs. Regarding our sampling strategy to gather experiences from at least 3 patients for each DMT, recruitment was successful (Table 1). Our analysis examined experiences of pwMS with DMTs and resulted in the identification of the themes shown in Fig. 1. All exemplary quotes which correspond to the themes can be found in Additional file 2.

**Table 1:** Demographic and MS-related characteristics of participants

Characteristic	N (%)
Females	35 (70)
Age (mean, range)	44.4 (21–61)
Highest professional qualification	
Still in vocational training	1 (2)
No professional qualification	2 (4)
Vocational education	27 (54)
Bachelor’s degree	4 (8)
Master’s degree, Diploma, state examination	14 (28)
Doctorate	2 (4)
MS type	
RRMS	44 (88)
SPMS	6 (12)
Years with MS since diagnosis (mean, range)	13.4 (2–33)
Patient determined disease steps (PDDS) (mean, range)	2.7 (0–7)
Current DMT use	39 (78)
Number of different DMT use so far	
0	3 (6)
1	3 (6)
2	11 (22)
3	13 (26)
4	12 (24)
> 5	8 (16)
Experience with DMTs	
Glatiramer acetate	16 (32)
Interferon-beta	33 (66)
Dimethyl fumarate	15 (30)
Teriflunomid	10 (20)
Alemtuzumab	7 (14)
Daclizumab	4 (8)
Fingolimode	17 (34)
Natalizumab	17 (34)
Cladribine	3 (6)
Ocrelizumab	8 (16)
Mitoxantrone	3 (6)

### Starting a DMT

#### Decision-making process

All participants had been engaged in decisions about DMTs. Forty-seven had experiences with at least one DMT. When we say “starting a DMT”, we do not refer solely to the first-time treatment with a DMT, but also to starting any new DMT. Different intercorrelated factors influenced the decision-making process when starting therapy with DMT.

The pwMS’ *autonomy preference in decision-making* differed, ranging from a physician-dominated, a shared, to a patient-dominated approach. PwMS who were faced with a decision right after diagnosis, reported a high level of helplessness, shock and trauma, and a lack of biomedical knowledge. Some pwMS, however, experienced a sense of relief that their symptoms now could be explained. Other felt powerless and overwhelmed by the amount of new information. In this situation, the physician’s recommendation had a high impact on the treatment decision: *“Unfortunately, you can quickly feel pushed into starting a therapy, that’s how I’d put it, because you’re immediately made to feel afraid, too. That you might end up in a wheelchair, say, that it will only get worse if you don’t do this. Yes, I felt really rushed into it, and that’s how I started out on therapies.” [pwMS 4].* Some physicians might have the tendency to motivate early treatment through anxiety.

On the other hand, our interviewees told how they were presented with information about a set of options by their physicians and were advised on all options. They used this information as well as other types of information sources, such as internet-platforms and information brochures, to prepare for a decision and informed choice at a follow-up appointment. Interviewees reported that they appreciated having an active role in decision-making. Thereby it was important to take breaks to process the information in order to be able to make a decision. However, there is a fine line as to whether patients see this as participation in decision-making or as being abandoned: “*But on the subject of therapy, he says: ‘So, you’re welcome to take these three brochures home, take a look and choose one.’ As an introduction, I thought that no bad thing. Then came the follow-up appointment, as agreed, and it was still: ‘Choose one.’ At that point I felt abandoned, not that he was giving me freedom.” [pwMS 3].* Patient-dominated decisions resulted from high autonomy preference in decision-making and occurred when switching or discontinuing treatment with a DMT.

Different *needs of the pwMS* turned out to be important factors in decision-making. One need was the desire to delay progression. Out of a sense of urgency pwMS wanted to start DMT immediately: *“And I thought, okay, maybe it’s not going to be so bad after all. That was my first relapse. And I’ve plenty of time. But nonetheless I should try to halt the disease course as quickly as I can.” [pwMS 33] – “Yes, of course at that time I was like: ‘Oh, just give me something to stop it getting too bad.’” [pwMS 2].* PwMS had the desire to do something about their situation especially after the diagnosis, take responsibility and start treatment to (re)gain control: *“It was really helpful having a therapy, something to actively combat the many fears that come into being at diagnosis time.” [pwMS 32].* Another need was a simple mode of administration of a DMT. If there was a choice between self-injectables, oral medication and infusions at the time of diagnosis, patients preferred oral medication or infusions.

After diagnosis, patients were faced with an *evaluation of possible benefits against possible risks of DMTs.* When the disease did not considerably influence everyday life, pwMS were encouraged to start with a less effective but low-risk DMT: *“Well, with the first-line therapies it was relatively straight-forward, even the word ‘first-line therapy’ isn’t too scary. The side-effects are straightforward […] [DMT B], yes, flu-like symptoms, that’s something you can cope with. […] [DMT H] was definitely a bit more difficult, because of that JC virus* [causing opportunistic infection of the central nervous system], *I wasn’t that old then, had all sorts of new plans. […] So really my choice was between continuing to suffer relapses and their unforeseeable consequences, or daring this leap.” [pwMS 16].*

During the process of starting a DMT, the *social environment* played a role, too. Firstly, there were the family and partners with whom patients consulted regarding DMTs. Secondly, there were other pwMS whose stories and views our interviewees sought when starting a therapy: *“At our MS group […] the consensus was actually: Yes, start a therapy right away. Any therapy is better than no therapy.” [pwMS 33].*

#### Effort in the mode and frequency of administration

PwMS experienced different forms of effort in the mode and frequency of administration in daily life as well as in the emotions and feelings triggered by handling DMTs. DMTs are available as tablets, syringes, or infusions. There were different views on which *administration form and procedure* was more convenient. For the majority, placing the syringe was a great challenge and many experienced injections as stressful and unpleasant. Some pwMS included their family in the administering of injections. By involving other people in the handling of certain DMTs, these may then be perceived as less amenable, because patients become dependent on other people: *“Before that I’d never had to give myself an injection. These are ones you just have to do subcutaneously into the stomach area, and my wife did the first one for me, but then for me it was a bother to ask someone else, so I learnt how to do it myself.” [pwMS 17].* On the contrary, for some pwMS self-injection was unproblematic.

For others, taking oral medications was more relaxed, although there is an increased risk that patients forget to take their pill: *“You take the tablets, one in the morning and one in the evening, which some people find tricky. It is easy to forget. That can happen reasonably easily when it is two tablets a day, but you get into the way of it, I do think. So, to begin with, I was always forgetting.” [pwMS 36].* The procedure around taking the pill, e.g. the strict handling instructions can be perceived as a burden. All in all, starting on oral medication was regarded as less intrusive. PwMS wanted a DMT, which does not confront them constantly with the disease. This was seen as the case for oral medication, but also infusions: *“The treatment [with DMT E] is in two subsequent years, in the first year you receive five infusions, and in the second year three infusions on three subsequent days, and in between there is no medication and that in itself is very pleasant.” [pwMS 48].*

Some pwMS preferred infusions to oral medications or syringes. Some described the infusion as relaxing and pleasant, because there was no need to think about taking it by themselves. Nevertheless, it makes a difference whether the infusion is monthly or half-yearly. Some patients stated that they felt well looked after in the clinic when integrated in an infusion scheme and during the infusions: *“This infusion is supervised throughout. […] So, I felt in really safe hands there.” [pwMS 48]*. But there were also pwMS who said that the infusion was stressful, unpleasant, and frightening. Some reported that the infusion gave them the impression of being more sick compared to oral medications, which are perceived as a more common part of everyday life: *“The moment I was lying there, whenever I entered, it was like that – yes, I have a serious illness. […] And that was different with the tablets. Because tablets are just much more integrated into the everyday.” [pwMS 34].* The interviewees described diagnostics and other procedures that must be carried out before starting therapy and during the treatment with DMTs – the determination of blood values, vaccinations and magnetic resonance imaging (MRI) scans, for example – as very time-consuming and burdensome. This may have made pwMS even more aware of the potential adverse effects of DMTs.

PwMS *developed a routine* for the self-management of a DMT: taking the oral medication with breakfast, or taking a warm shower before the injection to avoid haematomas. One patient reported how she takes time off when taking her DMT: *“Of course it means a week where the evenings are spent at home, I try to live more tranquilly. And definitely no alcohol that week. But it’s just a week, five evenings. And then it’s done. You take it in the evening. And that’s it, nothing more to it.” [pwMS 34].* Some forms of application were regarded as easily to be integrated into everyday life and others less so, when they had to be taken at a certain interval and in combination with food, for example.

Also, the complexity of handling a DMT in *work life* needs to be considered. Some DMTs demand a considerable amount of time for the mode of administration or thereafter. So, pwMS might need to take time off. Concerns have been expressed that time-consuming forms of administration may require an outing to the employer: *“I just don’t know how anybody with a full-time job who has fixed working hours and who doesn’t want their employer to know, I just don’t know how they do it. […] Perhaps I don’t have to say that it’s MS. But I do at the very least have to tell that I’m going to a doctor. And anyway you get a letter from the doctor as proof. And then any employer can see which [specialist] you’ve been to.” [pwMS 34].*

When *travelling*, it should be considered that some DMTs require cooling and that for injectables a certificate should be carried when traveling by plane, which reduces flexibility: *“In my job […] there were a lot of events to fly to. And it was quite an undertaking, getting through security with [DMT B], ensuring it remained well-cooled etc. The same with the needles going through security, quite a palaver too.” [pwMS 33].* Patients explained that they must prepare their vacation further in advance, especially when taking DMTs as injectables.

#### Success and failure of a DMT

For some pwMS, DMTs have not *effected on the relapse rate.* Others reported *reductions in relapse rates.* Some pwMS repeatedly reported *(supposed) improvements in MS symptoms*: *“Well, the advantage with [DMT H] was that symptoms that were residual from the last relapse, such as not being able to run, for example, disappeared entirely […]. And that’s when I actually completely forgot that I had the illness.”– “After my third infusion, I started to feel the effect, […] so much was well again, my walking was much better, I had a lot more sensation, yes, my body was calm again, [DMT H] had brought a peacefulness to the MS, had encouraged the healing, so to speak. There was so much that returned, became better, so much that you’d believed would never come back.” [pwMS 8]; “Started [DMT K], MS knocked on the head. It wasn’t there anymore, not even a ripple. It was gone. There wasn’t even the tiniest bit of activity that I was aware of. […] I could do all kinds of things, unrestricted.” [pwMS 11].* Although pwMS commonly attributed improvements in symptoms to the DMT, it might also be that the improvements occurred due to relapse remission or psychological effects.

#### Coping with adverse effects and failure of DMTs

DMT application is associated with adverse effects which pwMS experience and cope with differently. Some took *additional medication and therapies* and have not perceived this as a burden, e.g. anti-allergic drugs or analgesics against influenza-like symptoms. But a few have suffered serious adverse effects, which also required a more stressful therapy.

Some patients have *tolerated adverse effects* to prevent disease activity as “the price to pay”: *“My thyroid became overactive. You always think that won’t happen to me, those side effects won’t affect me. But unfortunately in my case, they did […]. Before I was diagnosed with the overactive thyroid, I just sat down on the floor and couldn’t get up. […] And it was clear when we started treating the thyroid it started getting better, a little better every week. […] But it’s all definitely better than being in hospital and sitting in a wheelchair […] I mean I was really ill before and now I feel great.” [pwMS 47].* Some pwMS also reported that they tolerate adverse effects which they did not perceive as very burdensome, such as hair loss, skin reactions at the injection site, flushes and gastrointestinal problems. Patients tolerate side effects if they feel stable and attribute this to the medication.

PwMS described *unusual adverse effects of DMTs*, which have not been reported by studies. Being aware that MS is the disease with 1000 faces, it is very challenging for patients to separate disease symptoms from other bodily sensations and side effects of DMTs. As people are always looking for causality, MS and DMT are first in line for such attributions.

### Switching to another DMT

#### Decision-making process

Experiencing adverse effects or no benefit were the major factors for the decision to change DMT. Adverse effects or DMT failure were either detected during a clinical examination and possibly in combination with paraclinical measure such as laboratory values or MRI imaging; or pwMS noticed them by themselves due to a reflection on new or worsening symptoms: *“That’s right, I took [DMT C] in the morning and just two hours later I had stomach pain, proper stomach cramps. I kept an eye on that for two or three days, because I thought it might just be a coincidence, could easily be something else. But it was always a couple of hours after taking a tablet that I started to feel ill, and then I…My neurologist was on holiday, sod’s law. I stopped taking them anyway without any consultation, for it seemed silly to take something that was causing me pain.” [pwMS 50].* The patients also reported that, although they had not experienced adverse effects during their therapy, they wanted or had to change their therapy because of anticipated adverse effects. Some interviewees did not want to accept constraints while still uncertain of the DMT’s benefits: *“But if I take medication that restricts me, and when I don’t know if it’s even going to work in the end or not, well, the MS itself is enough, I don’t need to add medication into the mix. That was how the decision to change […] came about.” [pwMS 17].* Some patients took a break from DMTs after experiencing no benefit from them and decided later to begin a new DMT if they noticed further disease progression, for example. Some of the pwMS had a desire to discontinue a DMT or at least take a break (“drug holiday”), but this was mostly rejected or not considered by the physicians as even a short interruption of treatment was deemed unacceptable and therefore pwMS had to switch to another DMT.

Again, there were personal *needs of pwMS* which were important in the decision to switch DMTs. PwMS who have had experience with DMTs, their handling and adverse effects, want a DMT that does not make them constantly think about MS in their daily lives: *“I was actually really happy when I got this. Not least because it meant an end to those weekly injections, the permanent confrontation with the illness. For this is quite different. You go in once a month and receive an infusion.” [pwMS 21].* The wish to have children also played a role in the women’s decision for DMT. Women who have taken a therapy not approved for pregnancy have to switch or interrupt it while trying to conceive. One woman explained that she preferred a DMT, which is administered in cycles as infusions, because it is approved for pregnancies after an appropriate period of waiting after the last cycle.

During the analysis, we noticed that the *social environment* was mentioned several times in relation to dealing with MS or handling injections. But it was hardly ever the case that pwMS said their significant others had any influence on the decision to switch a DMT. However, one pwMS reported that she changed her DMT not simply because of an adverse effect, but due to the responsibility for her children, too: *“I bear responsibility for my children. If I hadn’t had children, I would have probably stuck with [DMT H]. [pwMS 47].*

An *involuntary withdrawal of the DMT* was another factor for switching a DMT. PwMS described situations in which their previous medication was withdrawn, although they would have liked to continue it. A similar situation occurred for pwMS when they transitioned to SPMS and therefore switched to another DMT.

Some patients switched to a *newly launched DMT* by participating in studies, for example. The novelty of a therapy may not be the main argument, but it certainly aroused interest in this therapy and possibly was associated with the impression that a new therapy must be better than the old one. Conversely, one pwMS mentioned concerns about taking a recently approved new DMT: *“And then [DMT J] entered the market. Yes. And I thought to myself, I could give that a shot. But also, with some reservations, what with it being new on the market, what’s it like. I felt a little bit like a guinea-pig.” [pwMS 45].*

#### Coping with the decision to switch a DMT

Switching to another DMT evoked different *emotional reactions*. Some interviewees described this advocating a change in their DMT as a disappointment, especially when they did not perceive any adverse effects themselves, but laboratory abnormalities indicating organ dysfunction made this necessary: “*I experienced absolutely no side effects. I felt really great on it. I was also sad that I was feeling positive and yet had to stop taking it, otherwise I’d have stayed on it for sure.” [pwMS 47].* One interviewee described the process of switching DMTs as a repeated psychological burden, because the switch meant that the DMT had not been successful, leaving fewer options and the uncertainty of whether the next DMT will be successful: *“And so it was that feeling: okay, already one hasn’t worked and something new comes along and who knows if it will work? So that uncertainty and also disappointment, that was my feeling at the time.” [pwMS 36].* Several pwMS experienced relief at switching, because of the difficulty of administering the previous one or side effects.

44 PwMS have experienced at least two DMTs during their course of disease. There were eight people who have tried more than five DMTs. More than the half have experienced no treatment effect again, even after a change of DMT. Although therapy failed, pwMS *kept on going and proceeded with other DMTs*. One patient said that she was willing to try new DMTs over and over again and had confidence in physicians in this respect: *“And that is actually great that we have a few treatments to try out and there will be the one you decide to stick with and there will be one that can help.” [pwMS 15].*

### Discontinuing a DMT

#### Decision-making process

Eight pwMS discontinued their treatment and did not start a new one. The patients recounted that *no benefit of DMTs, adverse effects and burden in the mode of administration* were among their reasons why: *“It really was the case that the medication itself created more stress for me [doing the injections and the lipodystrophy as a side-effect] than the illness itself.” [pwMS 2].* Due to their negative experiences with DMTs, patients in this instance became critical of and felt a reluctance towards other DMTs.

One patient stated that she had stopped using a DMT because of the side effects, but also because she *possibly had benign MS*, having experienced one relapse in 10 years. Experience of the disease course over time may change patients’ attitudes towards treatment and may lead to discontinuation.

The *transition from RRMS to SPMS* was the criterion to stop the DMT for four pwMS. The reason was either a lack of accessibility to a DMT approved for the progressive MS course in the past or a limited number of DMTs remaining as an option.

#### Coping with the decision to discontinue treatment with a DMT

The decision against further treatment with DMT was associated with *uncertainty* because patients felt to bear a lot of responsibility within this decision: *“Yes, at the beginning, it is definitely strange. Is it the right decision or isn’t it? But as I mentioned, I did dedicate a lot of thinking time to it and no longer having to inject is a real relief. […] In that respect I felt good.” [pwMS 2].* Ultimately, the patient felt comfortable with the decision.

Some pwMS perceived DMTs as a threat and describe how their *well-being has improved* after discontinuing: *“I feel a sense of freedom.” [pwMS 4].* Other patients *defended themselves* when talking about their decision to stop treatment: *“I tried it out with several medication therapies, gave it my best shot, didn’t resist them.” [pwMS 17]; “I know from a friend of mine who is the same age as me, also has MS, a similar course […] I know, you can’t generalise, but although he did everything possible, tried out all possible medications, it got steadily worse right up to his death. Bearing that in mind, I really can’t say that I regret my decision about 15 years ago to stop taking any medication. It’s true, of course, that I can’t stand, that I can’t walk. But my head is still working well. And I am still alive.” [pwMS 29].* The latter pwMS justified his decision by comparing his disease state with that of his friend, and by doing so, critically appraised the benefit of DMTs.

For some pwMS, discontinuation meant *accepting MS and impairments* on a different level and enabling living more in the here and now: *“And now I haven’t taken any further medication for three years. […] I want to keep on enjoying life, just as it is. Sure, there are limitations […]. But not everything has to do with MS. That’s my experience at least. You have to learn to listen in deeply to your body, get to know yourself.” [pwMS 4].*

While having discontinued DMTs, the pwMS used *other therapy approaches* such as symptomatic treatment, corticosteroids, complementary and alternative medicines (CAMs): *“And if I do then have to deal with more severe relapses or problems, then I’d prefer to rely on a course of cortisone.” [pwMS 17]; “So I do other things […] I smoke joints, […] I take sulphur, […] I drink lots of ginger tea, […] I go more for the natural remedies.” [pwMS 4].*

### MS without starting a DMT

#### Decision-making process

Nine pwMS decided not to take a DMT directly after diagnosis and continued thus for many years. In our sample, there were three pwMS (pwMS 39, 40, and 42) aged 50–59 years, who have never undertaken treatment with a DMT.

Some pwMS initially decided against a treatment with DMT because there was *little or no choice of DMTs* years ago.

The patients’ *critical attitude towards conventional medicine and DMTs* was another reason not to start with DMT. One pwMS, who has never used a DMT, recounted: *“It is actually still not understood what actually causes MS, what the complicated interconnections are, and so on […] and then there are various drug therapies. […] But I found the very names scary, all the names these drugs have. Anyway, it was clear from the start there was no way I’d be taking any of them.” [pwMS 39].* The patient was additionally critical of DMTs because he did not consider the current state of MS research to be advanced. DMTs do not necessarily reduce relapses, and the long-term health benefit is not clear, which also causes feelings of uncertainty.

Another reason not to start a DMT is the *absence of a recommendation by a physician for a DMT*. One pwMS reported that because of this reason, she did not seek treatment with DMTs on her own: *“So, when it comes to these drug therapies, no one ever told me I should take something […]. And I had a neurologist, […] he said himself that he adhered to things that are statistically proven. But if I were to take a different path, that would be fine, too. But it was more at that level; he didn’t contradict me. […] And with no one insisting upon it, I stopped […] seeking medication and I simply did nothing.” [pwMS 42].* Other pwMS, who live without DMT said that a physician had even recommended not to start with a DMT: *“I should follow a healthy life-style. Shouldn’t smoke, and should do sport, and I shouldn’t eat too much pork. That was the long and the short of my doctor’s advice, rather than assigning me medication. [pwMS 18]; “Back then the doctor at hospital said, ‘Don’t give your body any substances, or it will get curious about them. So try to avoid them as long as you can’, and that was how I lived. […] I personally believed at the start that if I were to give my body something, then it would get used to it, and that might reduce the effectiveness of other mechanisms which are also important, and thus I told myself, no. As long as I am not restricted, then I won’t take anything.” [pwMS 40].* Here, the idea or approach of not accustoming the body to any substances seem to be present when reflecting on long-term medications.

A possibly *benign course of MS* and lack of perceived impairment caused by MS has led pwMS to decide against starting a DMT and not to do so for a long time: *“Because it had been only a brief exceptional event, a relapse, that I had back then, and it cleared up pretty much within four to six weeks, and no residual damage. […] Yes, so I was ill for four weeks and then… Yes, it was simply suppressed then, the MS. And for the first three years I basically didn’t take any medication because there was nothing wrong with me. I was healthy, as far as I was concerned. And the neurologist I had then, he also said: ‘You can take this, or not, as you like.’ And at that time, when I was diagnosed, there were only two therapies. […] And it was like that: ‘You can take it or not, either way. We don’t know whether it helps.’ Yes, and when you’re 18, 19, or 20, you think ‘I’m not interested!’ And get on with living.” [pwMS 23*].

#### Coping with the decision not to start treatment with a DMT

Patients described that they *felt comfortable* with their decision. One patient even felt lucky for not having been exposed to such a situation: *“It was a stroke of luck for my life that no one forced me to take anything.” [pwMS 42].*

PwMS took *other treatment approaches* such as relapse therapy, symptomatic treatment, as well as CAMs instead of a DMT. Using other therapy approaches makes pwMS feel (re)gaining control over the disease: *“So, I tried out various things […] for example: […]meditation. You don’t simply sit there listening to music and struggling with the thoughts that come into your head, but rather we visualise in our mind what the life we want to live looks like. For our brain cannot differentiate between what is imagined and what is actually taking place and if we give that a positive turn, then we send into our body all the good things it needs to be healthy […] feeling good rather than feeling the fearfulness of a victim’s state.” [pwMS 2].*

As mentioned before, pwMS who decided not to start a DMT seemed to be experiencing a benign course of MS with no noticeable impairment caused by MS. They moved forward with their life and were willing to *tolerate impairment* caused by the MS: *“Sometimes minor relapses occurred, slight distortions in sensation, but nothing that was really of major significance, and it was only over the course of two or three years that the regularity increased a bit. […] I saw them on MRI images, where it was always: ‘oh, here are a couple of bright circles, those were relapses.’ And then I’d say, ‘I hadn’t noticed, but if you say so, there must have been.’” [pwMS 40].*

Some people decided to wait for a time after diagnosis and not to take a DMT immediately. However, when people noticed increased disease activity, they then *start a DMT* in the hope of reducing MS progression and relapses.

## Discussion

In this article, we have given an insight into the experiences of pwMS covering four interrelated themes: (1) starting a DMT, (2) switching to another DMT, (3) discontinuing a DMT and (4) MS without starting a DMT.

Living with MS is characterized by experiences of uncertainty and the desire to (re)gain control over the disease, which was apparent in all the themes. As MS courses vary from no persisting impairment throughout a lifetime to early death, MS fosters a coping challenge of uncertainty which is unique among chronic diseases.

The decision-making process for or against a DMT and the use of those treatments can be understood as a constant, continually shifting process, complicated by different factors, which change over time in the MS course [10, 17]. Patients who felt powerless after diagnosis, in particular, preferred a more passive role in decision-making. However, our interviewees reported that they appreciated having an active role in decision-making from the very beginning, too. These findings are consistent with previous research on cognitive processes and experiences of pwMS engaging in treatment decsison making processes [5, 9, 10]. PwMS had the desire to do something about their situation especially after the diagnosis, to take responsibility and start treatment to (re)gain control as has previously been recognized in a phenomenological study on patients’ experiences with RRMS [18]. Tintoré [19] conducted a survey with 900 neurologists and 982 pwMS and state that patients’ satisfaction with a DMT is also related to the feeling of comfort in speaking with their physician and actively participating in their DMT decision-making. Moreover, the physician is challenged by estimating both the chances for success and failure of DMTs and disability progression. Therefore, both pwMS and physicians share this uncertainty. It is important for both to proceed step-by-step, talking about communication, and their way of communicating with each other – i.e. the way information is exchanged, what is to be communicated when and how decisions are reached.

When deciding for a DMT, one of the pwMS’ needs was a simple mode of administration. Whenever patients had the choice, they opted for oral medication. This is in line with the work of Jonker [20], who discovered in a discrete choice experiment that 42 % of pwMS prefer oral medication, followed by infusions (38 %), injections (16 %) and no DMT (4 %). Moreover, when relapses did not significantly influence pwMS’ everyday life, pwMS considered starting with a less effective DMT. More effective and riskier DMTs are more likely to be considered when relapses significantly affect daily living [9]. The social environment as well as other pwMS with their stories and views were important for decision-making. Ziebland and Wyke [21] describe in their realist review that when being faced with new health concerns or treatments, people search not only for fact-based information but also seek other patients’ experiences. Patients may gain feelings of control and confidence in facilitating self-management and coping with uncertainty while experiencing how others did manage. However, there are also concerns about the use of unbalanced and selective experiences due to the risk of conveying manipulative information.

After starting a DMT, patients made different experiences with the demands DMTs make on everyday life. The majority experienced injections as stressful and unpleasant, which is in line with other qualitative study work [6] and a cohort and cross-sectional study on adherence to DMTs in Germany [22, 23]. Oral medication was regarded as less intrusive, as also described by Van Reenen [18]. Some preferred infusions, because they did not need to apply it themselves and felt well looked after in the clinic. But there were also pwMS who reported that the infusion gave them the impression of being more sick compared to oral medications, which are perceived as a more common part of everyday life, a finding which has also been reported by [6]. Some newly diagnosed pwMS do not want to disclose their diagnosis to their employers and base their treatment decisions on how they may fit their work situation [9]. This contributes to the complexity of handling a DMT in everyday life.

Although DMTs aim to reduce the number and severity of inflammatory attacks and delay the onset of the progressive phase of MS, our interviewees not only experienced success and failure of DMTs, but commonly attributed (supposed) improvements in symptoms to the DMT. Eskyte [9] discuss that there is no consensus among pwMS on the definition of DMT efficacy. Asking patients for the experienced efficacy, especially as regards improvements, might thus help to understand DMT perception and help to better predict adherence.

After experiencing and coping with the effort in the mode and frequency of administration, adverse effects and failure of the DMTs as well as discovering personal needs (e.g. the wish to have children or bear responsibility for a family), pwMS became more active in the decision of switching or discontinuing a DMT. These findings have previously been recognized by a systematic review on pwMS needs and preferences when making treatment decisions [11], Visser [11] and cross-sectional studies evaluating DMT swtiches and adherence in German cohorts [24, 25]. A perceived lack of efficacy might be related to the occurrence of disease activity, but also to the substantial time gap between the act of taking the treatment and the desired outcome being in a distant future [9].

PwMS who decided to discontinue their treatment and did not start a new one, defended themselves when talking about their decision, which may come from an understanding “that taking action independently from the physician is considered a violation of cultural norms” [10], which can lead to feelings of guilt and enhanced uncertainty. On the other hand, some pwMS felt relieved and encouraged to become more conscious about their mind and body, and to enjoy life as much as possible and for long as possible without a DMT [18]. Based on these reported experiences physicians might actively address possible thoughts about stopping a treatment or having at least a drug holiday with at least a monitoring concept to avoid uncontrolled treatment stopping. The recently published new Germann MS treatment guideline [26] for the first time addressses this highly relevant issue from the patient perspective which is on the other hand highly ambivalent for treating neurologists. Hopefully the guideline will enable more open discusisons on this issue.

Decisions against starting a DMT were mostly patient-dominated, which is also found in other research [5, 10]. One reason for not starting a DMT was that pwMS were faced with this decision at a point in time when there was little or no choice among DMTs. Another reason for not starting a DMT was having quite a critical attitude towards conventional medicine. Moreover, relapses which do not influence the everyday life of pwMS significantly encourage pwMS not to start a DMT. Van Reenen [18] describe that pwMS do not start a DMT immediately, because as long as no symptoms are experienced and the body functions in the way pwMS are used to, they do not feel ill. As long as the disease is invisible to others, others do not regard them as ill persons. This represents another unique challenge in MS treatment handling: pwMS may have no symptoms for years but need to manage a regular medicaton with monitoring needs and side-effects.

Instead of a DMT, pwMS took other management approaches such as focussing on relapse therapy, symptomatic treatment, or CAMs. The latter, in particular, is a poorly researched area, lacking evidence on effectivity or safety [27]. Deciding against a DMT may free pwMS from worrying about risks, experiencing side effects and inconvenience [9]. However, when pwMS noticed increased disease activity, then they decided to start a DMT [18].

## Strengths and limitations

To achieve rigor of richness, we used the maximum variation sampling strategy [12], rigorous data collection and analysis methods such as the thematic analysis [16], and a traceable documentation of the analysis in MAXQDA Analytics Pro 2018. The collaborative multiprofessional team discussions at different steps of the study promoted transparency, multi-perspectivity and reflexivity [28]. The detailed description of the research process and participants enable the reader to assess whether the findings are transferable to other settings [29, 30]. Although recruitment was successful, finding participants who were willing to be video- and audio-recorded was challenging. Hence, we might have got a sample of pwMS whose personalities are characterised by higher openness, extroversion, and agreeableness than the “average” pwMS. The researcher conducting the interviews and analysing the data, is female and had no personal relation to MS, which could have influenced the relationship to the interviewees and the richness of the outcome in both directions, positively and negatively. Furthermore, the different interview settings (e.g. in a clinic or participant’s home) might have impacted the findings so some extent. However, the pwMS gave the impression that they wanted to tell their story and therefore reported in detail and honestly. Following the realist approach we acknowledge reporting an assumed reality evident in the data [16].

## Conclusions

MS is the disease with 1000 faces. 1000 faces are personifying 1000 opinions, belief systems, attitudes, emotional reactions, uncertainties – and decisions. PwMS are facing uncertainty and inconsistencies in science and knowledge about MS, complex evidence about what causes MS, how the disease might evolve and what the benefits of treatments are. This has an impact on their decisions (i) to start or not to start, (ii) when to start, switch or stop, and (iii) which treatment to take. PwMS interpreted DMTs as an opportunity, a threat or both depending on their constantly changing life circumstances, their MS and their previous DMT experiences. The findings demonstrate the importance for practitioners to look at current life circumstances of pwMS when recommending a DMT. Taking a DMT is a responsibility, not only for the patient, but also for the significant others. A possible approach is to give patients the opportunity to postpone the start of a DMT or to have a treatment break, if they are unsure and ambivalent until the point when they have reached more certainty. This in turn leads to the proposal to promote and enable pwMS to make informed decisions. This article provides the readers with a dense and rich glimpse of patients’ experiences with DMTs in daily life mirroring an attempt to handle uncertainty and to regain control and integrate adaptivity into one’s life.

## Supplementary Information

**Additional file 1.** Interview guide.

**Additional file 2.** Themes, sub-themes and the corresponding exemplary quotes from pwMS.

## Data Availability

The data generated and analysed during the current study are not publicly available due to privacy issues but are available from the corresponding author on reasonable request.

## Abbreviations

CAM: Complementary and alternative medicine
DMT: Disease-modifying therapy
MRI: Magnetic resonance imaging
MS: Multiple sclerosis
PDDS: Patient Determined Disease Steps
PPMS: Primary progressive multiple sclerosis
pwMS: People with multiple sclerosis
RRMS: Relapsing-remitting multiple sclerosis
SPMS: Secondary progressive multiple sclerosis
