# Supplementary material for: Patients experiences with multiple sclerosis disease-modifying therapies in daily life – a qualitative interview study
Source: BMC Health Serv Res. 2021 Oct 22;21:1141. doi: 10.1186/s12913-021-07012-z (PMC8539738; doi:10.1186/s12913-021-07012-z)
Supplement: Supplementary file 1 — Additional file 1. Interview guide. [file 12913_2021_7012_MOESM1_ESM.pdf]

Patients Experiences with Multiple Sclerosis Disease-Modifying Therapies in Daily Life – a Qualitative Interview Study

Anna Barabasch[1*], Karin Riemann-Lorenz[1], Jutta Scheiderbauer[2], Ingo Kleiter[3], Rebecca Morrison[4], Christopher Kofahl[2,5, **], Christoph Heesen[1,6, **]

[1]Institute of Neuroimmunology and Multiple Sclerosis (INIMS), University Medical Center Hamburg-Eppendorf (UKE), Hamburg, Germany. [2]Patient representative. [3]Marianne-Strauß-Klinik, Behandlungszentrum Kempfenhausen für Multiple Sklerose Kranke gGmbH, Berg, Germany. [4]freelance author. [5]Institute of Medical Sociology, University Medical Center Hamburg-Eppendorf (UKE), Hamburg, Germany. [6]Department of Neurology, University Medical Center Hamburg-Eppendorf (UKE), Hamburg, Germany.

** Joint senior authorship

*Corresponding author at:

Institute of Neuroimmunology and Multiple Sclerosis
University Medical Center Hamburg-Eppendorf
Martinistrasse 52
20246 Hamburg
Germany
Email: a.barabasch@uke.de
Tel.: +49 (0)40 7410-53215

Supplementary Info File

Additional file 1. Interview guide

Guiding questions	Check: Was that mentioned?	Specific questions
I. I would like you to tell me about your life with MS. The best way to begin is to start with how you were diagnosed with MS.<br><br>You can take your time, even for details, because everything that is important to you is interesting to me.	• Symptoms and indications before diagnosis<br>• Medical examinations<br>• Diagnosis	
II Please tell me if you have informed people around you that you have MS.<br><br>If so, what was it like when you told them?	• Who has the pwMS told that he or she has MS?<br>• When did the pwMS tell about MS?	• How did you tell those in your social environment?<br>• When did you tell them?<br>• Who did you tell?<br>• Who were you worried about whether or not to tell about your illness?
III. Please tell me about your experience with DMTs.	Which DMTs were used?<br><br>• Glatiramer acetate<br>• Inteferon-beta<br>• Dimethyl fumarate<br>• Teriflunomide<br>• Alemtuzumab<br>• Daclicumab<br>• Fingolimod<br>• Mitoxantrone<br>• Natalizumab<br>• Cladribine<br>• Ocrelizumab<br><br>Handling and side effects	Can you tell me how it was for you when you had to decide on a DMT or against a DMT?<br><br>Question to pwMS who have taken DMTs:<br>• Have you had any problems with the DMT?<br>• Can you tell me how you are taking/took this medicine and how you are coping/coped with it?<br><br>Question to pwMS who have not taken DMTs:<br>• How did you feel about not taking a DMT?<br>• Did you have any problems with not taking a DMT?

IV. Apart from DMTs, there are other therapies that can be used. These include alternative therapies or measures that change lifestyle habits.<br><br>If you have tried anything, please tell me about your experiences with it.	Alternative therapies, e.g. homeopathy, acupuncture, treatments on biological basis<br><br>Lifestyle measures, e.g. nutrition, exercise and sports, relaxation and stress management	• What have you already tried out and how did you experience it?<br>• How did you decide to do this?
V. What experiences have you had with rehabilitation methods?	Outpatient rehabilitation? Inpatient rehabilitation?	If rehabilitation was done:<br>• Which impact did the rehabilitation have on you?<br>• How did you decide to do rehabilitation?<br><br>If no rehabilitation was done:<br>• Was that a topic of discussion at any point?<br>• If so: Can you tell me about how you decided against rehabilitation?
VI. Could you please tell me about your everyday life with MS.	• Work life; disability and retirement<br>• Social life, stigmatisation<br>• Pregnancy, birth, children<br>• Adaptation and aids<br>• Mobility, Travel<br>• Nutrition, food supplements<br>• Exercise, sports<br>• Relaxation / stress management	
