# Supplementary material for: Patients experiences with multiple sclerosis disease-modifying therapies in daily life – a qualitative interview study
Source: BMC Health Serv Res. 2021 Oct 22;21:1141. doi: 10.1186/s12913-021-07012-z (PMC8539738; doi:10.1186/s12913-021-07012-z)
Supplement: Supplementary file 2 — Additional file 2. Themes, sub-themes and the corresponding exemplary quotes from pwMS. [file 12913_2021_7012_MOESM2_ESM.pdf]

Additional file 2. Themes, sub-themes and the corresponding exemplary quotes from pwMS

**Themes and sub-themes**	**Main quotes**
Starting a DMT\Decision-making process\Autonomy preference in decision-making\Physician-dominated approach	There was no decision-making process, I asked what I could do, at that moment you're totally overwhelmed by all the information, not being a doctor, not educated in this. [pwMS 22]
	Unfortunately, you can quickly feel pushed into starting a therapy, that's how I'd put it, because you're immediately made to feel afraid, too. That you might end up in a wheelchair, say, that it will only get worse if you don't do this. Yes, I felt really rushed into it, and that's how I started out on therapies. [pwMS 4]
Starting a DMT\Decision-making process\ Autonomy preference in decision-making\Shared approach	The reason I really like my doctor is that he has always let me be the one to decide. He has always said to me, right, these would be our next options. This medication is not an option for this and this reason. And that medication he would caution against because. Until eventually two or three medical options remain. And he'd go through the advantages and disadvantages of each. Then I would go home and do some more research of my own, sit down with my boyfriend and with my family and consider which was the best match. Which risks was I willing to accept, or not. And the form of therapy. Did I prefer pills, did I want to keep injecting, and so on. And then I'd make my own decision. [pwMS 34]
	But on the subject of therapy, he says: "So, you're welcome to take these three brochures home, take a look and choose one." As an introduction, I thought that no bad thing. Then came the follow-up appointment, as agreed, and it was still: "Choose one." At that point I felt abandoned, not that he was giving me freedom. [pwMS 3]
Starting a DMT\Decision-making process\ Autonomy preference in decision-making \Patient-dominated approach	Then it turned out over the next few years, there was always one relapse. A single relapse and usually connected to times of intense emotion. And then, of course, at some time or other came the suggestion: "You should think about it", as they put it. And my reply was, I've been thinking about it for years, and I don't want to. Yes, that was my mantra back then, too: "No, I don't want to." [pwMS 31]
Starting a DMT\Decision-making process\Needs of the pwMS\Desire to delay progression	And I thought, okay, maybe it's not going to be so bad after all. That was my first relapse. And I've plenty of time. But nonetheless I should try to halt the disease course as quickly as I can. [pwMS 33]
	Yes, of course at that time I was like: "Oh, just give me something to stop it getting too bad." [pwMS 2]
Starting a DMT\Decision-making process\Needs of the	That was out of the question. It is probably to do with how I was brought up, the idea that if you are ill, you need medicine… probably a similar reaction to vaccination. It is important, so you do it, that's why it's there. [pwMS49]

pwMS\Desire to control the disease	It was really helpful having a therapy, something to actively combat the many fears that come into being at diagnosis time. [pwMS 32]
Starting a DMT\Decision-making process\Needs of the pwMS\Desire of a simple mode of administration of the DMT	I knew I didn’t want to inject myself every day, nor three times a week. Yes, that was quite important to me, knowing that would place too many constraints on life. So, my first-line therapy was [DMT B]. That’s once a week, into the muscle. [pwMS 27]
	So, he started listing my possible options, and we were straight into self-injecting with something or other. And that was simply a no-go option for me and I was really starting to think, no, I’d prefer just to be ill then and not take any medicine. No, injecting twice or three times a week, or whatever it is, that was simply not an option. That would be incredibly restrictive, I thought. No, I didn’t want to. And then he said, ah, but there is a pill form, too […], and I agreed to that. For me that was definitely the optimal choice. [pwMS 6]
Starting a DMT\Decision-making process\Evaluation of possible benefits against possible risks of DMTs	And it’s all a weighing up of risks. Whether this therapy or that is a decision about how willing I am to risk more serious side-effects in order potentially to have a more powerful effect. And is there any middle ground at all. And at the end of the day, that’s a personal decision for each of us. […] I was quite simply afraid of more serious side effects and thought, okay, that might be less bad with Beta. Maybe [DMT B] will be strong enough. [pwMS 33]
Starting a DMT\Decision-making process\Social environment	At our MS group […] I simply asked the others: How do you deal with it? How do you live with it? Are there things you had to learn the hard way? Are there Lessons Learned? Are there things you shouldn’t have done? The consensus was actually: Yes, start a therapy right away. Any therapy is better than no therapy. [pwMS 33]
	Then I went home and did some more research of my own, sat down with my boyfriend and with my family and considered which was the best match. [pwMS 34]
Starting a DMT\Effort in the mode and frequency of administration\Administration form and procedure \Self-injectables\Injections as stressful and unpleasant	It was difficult for me, because beforehand I would never have considered self-injecting. I’m no fragile flower, but thrombosis injections, for example, it was always a nurse who did those and self-injecting is something I’d never imagined doing, and I found it really difficult, oh yes, I really struggled at the beginning. [pwMS 48]
	[DMT B] is a weekly injection into the muscle. Usually I injected my thighs. It takes a little bit more will-power, for you’ve got to get it right into the muscle tissue. [pwMS 29]
Starting a DMT\Effort in the mode and frequency of administration\Administratio	For me the issue of self-injecting was no big deal. You disinfect the area and then you have the choice of either injecting manually or using the injecting device. I did both. I actually found the manual injection more pleasant than the automatic device. [pwMS 33]

n form and procedure \Self-injectables\Injections as not problematic	The actual injecting, that was absolutely fine, easy. [pwMS 13]
Starting a DMT\Effort in the mode and frequency of administration\Administration form and procedure \Self-injectables\Including other people in the procedure	In 2000, I started injecting. Back then you still had to be given the injection because it had to be injected right into the muscle. It was my daughter who said, "Listen, I'll come with you to the doctor', and I'll inject it into your buttocks, no problem." And so she learnt how from the nurse. And not long after that my husband decided the same. He said, "She's surely not the only person who can do it. We want to be able to go away on holiday or wherever. And rather than keep on going to the doctor, I'll learn how." And my husband did them from then on. [pwMS 19]
	Before that I'd never had to give myself an injection. These are ones you just have to do subcutaneously into the stomach area, and my wife did the first one for me, but then for me it was a bother to ask someone else, so I learnt how to do it myself. [pwMS 17]
Starting a DMT\Effort in the mode and frequency of administration\Administration form and procedure \Oral medications \Oral medications as relaxing and pleasant	And I just felt like a normal person, without an illness, just someone who had to take a few pills. […] And because it was so early, still half-asleep when taking them, you didn't have that image in your head – yes, you are sick, you have to take a pill every day. I didn't have that sense at all. And with Tysabri®, that is how it was. The moment I was lying there, whenever I entered, it was like that – yes, I have a serious illness. I am having this liquid, whatever it is, put into me intravenously. And that was different with the pills. Because pills are just much more everyday. [pwMS 34]
	And with injections you've always got this: Oh God, it's time for yet another injection. That was totally different with the pills. With pills, you don't feel half as ill. That's how it is for me, personally. I say to myself: okay, so I take pills, so what? I take other pills, too. [pwMS 30]
Starting a DMT\Effort in the mode and frequency of administration\Administration form and procedure \Oral medications \Oral medications as stressful and unpleasant	And then, yes, [DMT I] came along. And that's what I took. Exactly, that's also in the form of a pill, two months of the year, for a week each time, either one pill daily, or two. Depending on your weight, I think. Super to administer. What I found a little odd, personally, is that the instructions in the packet that you shouldn't put the pill down on any surface and that if you do, you have to wipe the surface carefully, and having held it in your hand, you should wash your hands carefully. I found that really strange, I am not supposed to put it down anywhere, or it's better not to, nor hold it in my hand, but it's ok to swallow it. [pwMS 50]
	[DMT I] are these really small pills which have this crazy packaging. […] you have to take the pills really quickly and carefully. You're not supposed to put them down anywhere. And then you have to use disinfectant, like it's some kind of highly toxic substance. Even my doctor says it's a bit over the top what's written on the packet or in the instructions inside. It's more like, sure, you shouldn't leave them lying around, of course, but it's not like there would be an explosion

	or anything if you did, or if you were wiping it clean, or anything, nothing would happen. [pwMS 34]
Starting a DMT\Effort in the mode and frequency of administration\Administration form and procedure \Oral medications \Risk of forgetting to take the pill	You take the pills, one in the morning and one in the evening, which some people find tricky. It is easy to forget. That can happen reasonably easily when it is two pills a day, but you get into the way of it, I do think. So, to begin with I was always forgetting. But in the meantime, that no longer happens. [pwMS 36]
	Yes, so first thing in the morning, when I'm brushing my teeth, that's when I take my pill. All in all, I have only forgotten once or twice. [pwMS 35]
Starting a DMT\Effort in the mode and frequency of administration\Administration form and procedure\Infusion\Infusion as relaxing and pleasant	Well, [DMT H] was super. The infusion, I just sat there and was given it. It was really easy, much easier than the pills, when it was up to yourself to remember them. So, I found infusions much better than pills. [pwMS 47]
	Nor did I have any problems with infusions. That was relatively quick, too. Here at the UKE it took about 15 minutes or so. And then I could go home already. The only slightly annoying thing maybe is that it's every month. You just have to factor it in. But I do find you get used to it. It becomes normal, going there every month. Although sometimes you can't quite believe another month has passed already. [pwMS 45]
Starting a DMT\Effort in the mode and frequency of administration\Administration form and procedure \Infusion\Feeling of being well looked-after	As far as I remember, there were no problems. It was the most pleasant form of therapy thus far, also because of the all-round service. […] The fact that you were at your neurologist's all the time, even now … he'd always look in, check everything was okay, so that if there been anything wrong, you really could give monthly feedback or ask questions. I really liked that. [pwMS 49]
	This infusion is supervised throughout. That in itself was a really good feeling, because you know, you read about it, but it's only later on that you become conscious of just how major it is, what's happening in your body and all that could happen […] So, I felt in really safe hands there. [pwMS 48]
Starting a DMT\Effort in the mode and frequency of administration\Administration form and procedure \Infusion\ Infusion as stressful and unpleasant	So, [DMT E], I found it quite a procedure, the infusion, you start in the morning with an infusion of Phenestil, then I think it was cortisone after that, and then you're on the drip for six hours, as it slowly goes in. The first days I was lying there hooked up to a heart monitor, just in case, I found that really tiring, but then you think it's alright, it's not forever, a week later it's all done, so I didn't find it too bad. [pwMS 7]
	It always frightened me, to be honest, because the side-effects are quite something, and the way it's administered is, you're an in-patient for five days, you're on a drip for ten hours there, you're given cortisone, and then the medicine that pretty much destroys your immune system, so you arrive healthy and you leave sick, if I can put it like that. [pwMS 16]

Starting a DMT\Effort in the mode and frequency of administration\Administration form and procedure\No constant confrontation with the disease	And I think that it gives you back a kind of quality of life when for six months you don't have to think about the fact that you have MS. I mean, unless you're feeling any odd sensations, that is. So, for me, when I'm feeling well, I really do forget about it. And I only notice it again when six months have passed. And so, for me it is absolutely fine to have to sit there for six hours for one day if it means I don't have to go there every month. That's relaxed. [pwMS 45]
	And then they changed me onto [DMT G]. And that was the best possible therapy for me, because it is a pill, that you take every day, just like the [contraceptive] pill. So, it hardly restricts you at all. You just have collect the prescription every three months and are left in peace otherwise. So, you can almost forget about it. It's there, but it is not restricting you. [pwMS 27]
Starting a DMT\Effort in the mode and frequency of administration\Administration form and procedure \Diagnostics and other procedures before and during a DMT	There's a certain amount to organise in the lead-up to it, first and foremost all those vaccinations. Looking for your vaccine pass, finding it, fixing appointments for the vaccinations, and then there's the examination ahead of the infusion itself, what is your vaccine status, or how you're doing with the infusions, and gosh, all those things that have to take place. It's quite time-consuming. So, it's not simply a case of turning up at the hospital, and being given [DMT J] by your neurologist, but instead you go to your doctor a couple of times beforehand, and also to the radiologist, to get brain-scan images, and so on and so forth. You have to dedicate time to it. It's a case of management of your illness in this context. [pwMS 44]
	And then I was able to join the final phase of the clinical study of the compound with Novartis. That was at the university clinic in Freiburg and my taking part depended on a number of things. My blood count, for example, no previous liver or kidney disease. My heart had to be in good order. My eyes, too, and so on. Just because, well… [DMT G] is a great medication but it carries a risk of some potentially serious side effects. And they have to be carefully watched for, which is why the first time it is administered there is strict medical surveillance. For that I had to spend an afternoon, an evening in the hospital, in the clinic, not overnight, but under observation for 10 hours. And it all went well. So, I was accepted onto the study. Blood tests every four weeks after that, check-ups to attend. [pwMS 26]
Starting a DMT\Effort in the mode and frequency of administration\Developing a routine	There is a plan telling you how many you should take. Just once a day. Best taken, of course, at the same time of day, but it doesn't have to be at a particular hour. Nor does it have to be in combination with a meal. And I find it's something you can really easily, or that I can, integrate really well into my life. Of course, it means a week where the evenings are spent at home, I try to live more tranquilly. And definitely no alcohol that week. But it's just a week, five evenings. And then it's done. You take it in the evening. And that's it, nothing more to it. [pwMS 34]
	I found [DMT C] a bit difficult in my case, integrating in into my every day, one pill in the morning, another in the evening, twelve hours in between. And now, well, I work full-time, I took one pill in the morning at work, but I found it quite difficult, I had to set an alarm clock, and if there was a meeting then or an out-of-house appointment, it wasn't ideal.

	And in the evenings, too, there are some days I go to bed at 8pm, because I'm so tired, but at 9pm it's time for the pill, so for me it was a bit tricky integrating it into my everyday. [pwMS 16]
Starting a DMT\Effort in the mode and frequency of administration\Handling DMT in work life	I just don't know how anybody with a full-time job, who has fixed working hours, and who doesn't want their employer to know, I just don't know how they do it. I mean, sure, you get time-off. But then I have to tell them that there's a therapy I need to get once a month. Perhaps I don't have to say that it's MS. But I do at the very least have to tell that I'm going to a doctor. And anyway, you get a letter from the doctor as proof. And then any employer can see *which [specialist] you've been to*. So, it's very hard to keep secret. [pwMS 34]
	The administering of the infusions was always good. I usually went to an outpatient tumour centre. I was given an infusion once a month. Timing-wise it was always fine to arrange. It meant taking a day off work once a month. My employer took all that on board, the fact that I had to get an infusion at the hospital once a month. I didn't have to make up time or anything. That was always a great benefit. [pwMS 25]
	I actually got on very well with [DMT H]. The only thing was, what with working full-time still, booking the time off to go to the neurologist, to lie there, get the treatment, that was always a bit difficult for me because my employer didn't know what I had. I mean he could see that there was something wrong with my walking, but he didn't know why, so… that was the stressful part of it. [pwMS 7]
Starting a DMT\Effort in the mode and frequency of administration\Handling DMT when travelling	So, I plan holidays quite carefully. I make a to-do list and depending on how long I'll be away, I look out either a big cool bag, or a smaller one. I have got special containers for my Glatiramer-acetate syringes. The thing is they shouldn't freeze, shouldn't be frozen. That's really important. When I'm on a bus journey I usually ask the driver if I can put my syringes in the drinks fridge. Most of them don't have a problem with that, what with them being in a separate container. The actual syringes aren't visible. And if I'm travelling by car, I take my cool-bag and keep my cool-box in that. I've never flown with Copaxone injections. The thing is, you can actually keep Copaxone out of the fridge for 14 days. When you're travelling abroad, you require a medicine pass, because there's always the possibility that your medication isn't known there. There's an appropriate pass for each country with the appropriate written explanation, so that it's clear what the medication is for. [pwMS 28]
	In my job […] there were a lot of events to fly to. And it was quite an undertaking, getting through security with [DMT B], ensuring it remained well-cooled etc. The same with the needles going through security, quite a palaver too. And even if you have the certificate saying it's alright, it's opened up and looked at anyway. That means that all the flexibility that is part of travelling for business, just with hand luggage, getting through check-in quickly and then directly onto the flight, which we had down to a fine art, well, all that was a thing of the past for me. [pwMS 33]

Starting a DMT\Success and failure of a DMT\ No effect on the relapse rate	It actually agreed with me really well, and I experienced practically none of the side effects that are listed, and the actual administering of it, that was absolutely fine, too, and I actually always really believed in the effectiveness of that medication and it was all fine for me, except unfortunately then, whilst on Tecfidera®, I had a whole series of relapses. [pwMS 48]
	I started with [DMT A], which you inject every second day. That is the slightly higher dose. Unfortunately, it didn't really kick in for me. After a year, my results were still very poor. There were still lesions in my brain. The MRI didn't show any real improvement. And then I was taken off it. [pwMS 43]
Starting a DMT\Success and failure of a DMT\ Reduction of the relapse rate	And it was indeed an enormous change. Right from the start I had the feeling that it was really having a big impact. At that time, I had just finished my studies and had started work. And my first job was a really stressful one. Sometimes I was working 60 or 70 hours a week, so not really what you're advised to do with a chronic illness, but with [DMT G] it was no problem. Actually, I have to say that I think that [DMT G] phase, which went on for about five years, was the fittest period of my whole life. I felt so good. Initially I had no obvious side effects at all. I had one relapse that entire time. [pwMS 36]
	And I have to say with hindsight that it was an inspired decision to take [DMT A]. So, I took [DMT A] from 2003 for almost ten years through to 2013. And I had a reduction in my relapse rate: from one or two relapses a year to almost half that. I experienced a relapse then only every second or third year, and then it was a minor relapse. By that I mean they weren't as serious as that first one when half of my face was paralysed, instead I had pins and needles or altered sensation, things like that. And that was all. [pwMS 23]
Starting a DMT\Success and failure of a DMT\(Supposed) improvements in MS symptoms	And [DMT H] worked brilliantly for 38 months. I didn't have any relapses. I must tell you, I went from being a wheelchair-user to being able to walk again. It was incredible. I could cycle really well, was truly back in business, my concentration was excellent. All that had been really, really difficult before. So Tysabri had given me so much quality of life, but then it had a terrible side effect, and no one wants that. [pwMS 28]
	Started [DMT K], MS knocked on the head. It wasn't there anymore, not even a ripple. It was gone. There wasn't even the tiniest bit of activity that I was aware of. […] I could do all kinds of things, unrestricted. Without consequences. [pwMS 11]
	After my third infusion, I started to feel the effect, my body came to rest, I noticed it could heal, so much was returned to me, so much was well again, my walking was much better, I had a lot more sensation, yes, my body was calm again, [DMT H] had brought a peacefulness to the MS, had encouraged the healing, so to speak. There was so much that returned, became better, so much that you'd believed would never come back. [pwMS 8]

Starting a DMT\Coping with adverse effects and failure of DMTs\Additional medication and therapies	I got quite a temperature, too. At least once or twice a month I also got the shivers at night and a 40 degrees fever. This in spite of the 1g of Paracetamol I took the whole year through. [pwMS 32]
	[DMT H] is a wonderful medication, except it has one terrible side effect and that is progressive multifocal leukoencephalopathy (PML) […] The treatment for PML is very, very tough. You are given plasma transfusions, which means, you get a complete blood exchange over the course of five days. That means you are given donated plasma, your own blood is got rid of. [pwMS 28]
Starting a DMT\Coping with adverse effects and failure of DMTs\Tolerating adverse effects	I've been taking this [DMT D] for about half a year now. And in the first two months the side effects were very, very, very tough! I was so close to stopping because it was simply unbelievable. It was really… the hot flushes were the least of it, but…I was totally exhausted, couldn't do a thing, couldn't really move properly. Well, it was really tough for the first two months, but then at some point, that went away. And then a couple of months after that, my hair started falling out. That wasn't so nice either, suddenly being half-bald. Now the hair has started to come back a bit, but that was it, and now I swallow them down like sweeties. There aren't any side effects at all now. [pwMS 35]
	My thyroid became overactive. You always think: that won't happen to me, those side effects won't effect me. But unfortunately in my case, it did [,,,] Before I was diagnosed with the overactive thyroid, I just sat down on the floor and couldn't get up. I just didn't have the strength, I couldn't climb the stairs at all. First, we thought it was a relapse, but then it turned out to be an overactive thyroid. And it was clear when we started treating the thyroid it started getting better, a little better every week. […] But it's all definitely better than being in hospital and sitting in a wheelchair […] I mean I was really ill before and now I feel great. [pwMS 47]
Starting a DMT\Coping with adverse effects and failure of DMTs\Having unusual adverse effects	It was only when I stopped taking them that I realised what the pills had been doing. It was only then that… my body changed in a way that felt unbelievably good to me, and somehow kind of lifted a burden from me. Looking back, I think the [DMT G] settled on my lungs somehow, so I kept having a sensation of pressure on my lungs, which I hadn't associated with the pills. [pwMS 36]
	I got these herpes blisters every time, which apparently lots of people get with [DMT H]. [pwMS 28]
	[DMT C], yes, I had the lower dose, which went well for the first week. But when I started taking the higher dose, I got ulcers on my tongue and stopped taking it then. [pwMS 4]
Switching to another DMT\Decision-making process\No benefit from DMT	After that came [DMT C]. Those are pills, again to be taken every day. It was clear pretty quickly, on my second day, I think, that [DMT C] and I weren't getting along. That's right, I took it in the morning and just two hours later I had stomach pain, proper stomach cramps. I kept an eye on that for two or three days, because I thought it might just be a coincidence, could easily be something else. But it was always a couple of hours after taking a pill that I started to feel ill,

	and then I…My neurologist was on holiday, sod's law. I stopped taking them anyway without any consultation, for it seemed silly to take something that was causing me pain. [pwMS 50]
	Then I had to simply stop because it is limited to twelve or twenty-four months, after that a JC virus can occur in the brain. Yes, in fact I did miss it, I had got along with it very well, had been totally satisfied with it. After that I returned to [DMT G] in the hope that the quietness my body had experienced in those last months would be helpful here, too. But that unfortunately was not the case. And thus, I arrived at my current medication, [DMT E]. [pwMS 16]
	But if I take medication that restricts me, and when I don't know if it's even going to work in the end or not, well, the MS itself is enough, I don't need to add medication into the mix. That was how the decision to change […] came about. [pwMS 17]
	Well, I did consider from time to time how it would be to stop taking any medication at all and to declare that I would rather live my life without medication. But then from my own side there was fear about the possible rebound effect from [DMT H]. And that it all might be worse than before. And I wasn't keen on that. And then I had also heard from the doctor's perspective that this risk was certainly not to be underestimated, and that it would definitely be better to continue. [pwMS 45]
Switching to another DMT\Decision-making process\Needs of pwMS\No constant confrontation with the disease	I was actually very happy when it came because I'd heard such good things about what helpful medication it was, how it drastically reduced the risk of relapse, and improved mobility, and so I was actually really happy when I got this. Not least because it meant an end to those weekly injections, the permanent confrontation with the illness. For this is quite different. You go in once a month and receive an infusion. [pwMS 21]
Switching to another DMT\Decision-making process\Needs of pwMS\Wish to have children	Then I was given [DMT H] for a year, or a year and a half. […] During that time I experienced no relapses, yet there is always the concern that you can't take it for too long, and so I stopped taking it when thinking of pregnancy, of the desire for a child, the child I wished-for. And then it so happened that after pregnancy I had quite a severe relapse, and could see almost nothing out of the left side, then major double-vision and when you can't see your own child, it is frightening, it really is. Into the hospital then, […] a consultation, and Lemtrada® was recommended, and I gratefully accepted it and have been happy with it ever since. [pwMS 49]
	I haven't had any treatment since May of last year, that was when I received my second batch of [DMT E], that meant two days in hospital. […] I feel healthy, I could have children now, too, I didn't have to put anything aside, and that was actually one of the reasons why I chose it. [pwMS 16]
Switching to another	I experienced absolutely no side effects. I felt really great on it. I was also sad that I was feeling positive and yet had to

DMT\Decision-making process\Social environment	stop taking it, otherwise I'd have stayed on it for sure. My actual thinking was: I bear responsibility for my children. If I hadn't had children, I would have probably stuck with [DMT H]. [pwMS 47]
	I talked it through with my husband, I spoke with friends and family, colleagues, that's to say colleagues from my honorary post and with my doctor, of course. And at each of our yearly check-ups I broached the subject, and, yes, gradually there was something else that had been approved. [pwMS 3]
Switching to another DMT\Decision-making process\Involuntary withdraw of the DMT	Yes, I switched to [DMT J] in 2018. And that was the advice of the doctor at the clinic, that we could assume that I was at the age where the shift towards the progressive form of MS could happen, that it could gradually take over from the relapsing variant. [pwMS 25]
	So, I took [DMT F] over a relatively long period and we were a great match. It was fantastic. It was the very thing for me, and I thought to myself I could take it until the end of time. Unfortunately, however, somebody came between us, saying the medication was being withdrawn from the market. Because they no longer felt they could take the risk of some people dying from inflammation of the brain. But that's just how it is, right? What works brilliantly for some, is sub-optimal for others. At any rate, I had to stop taking it then. [pwMS 38]
Switching to another DMT\Decision-making process\DMT new on the market	And then [DMT J] entered the market. Yes. And I thought to myself, I could give that a shot. But also, with some reservations, what with it being new on the market, what's it like. I felt a little bit like a guinea pig. [pwMS 45]
	And then after consulting my neurologist I decided to try [DMT C] because it was being praised as the gold-standard treatment in the United States and in Germany, too, it was praised in various presentations again in the same media, Amsel and co., as a medication that was well-tolerated. [pwMS 22]
Switching to another DMT\Coping with the decision to switch a DMT\Emotional reactions	Anyway, at some point [DMT C] also stopped being effective. And then we decided, it was almost exactly a year ago, so the autumn or winter, no it was the autumn of 2017, to stop. I was really, really relieved about that. I mean, I'd already been relieved when I stopped [DMT B] and [DMT H], but I was really relieved when I could stop taking the [DMT C] pills. [pwMS 34]
	And so, it was that feeling: okay, already one hasn't worked and now something new comes along and who knows if it will work? So that uncertainty and disappointment, that was my feeling at the time. [pwMS 36]
Switching to another DMT\Coping with the decision to switch a DMT\Keep on going and	And then we came onto my fifth treatment, and my last one to date. It is [DMT I]. […] It had been approved once before in Germany. But unfortunately, there had then been a death. And then of course it was taken off. But now it has quickly been approved again. And it is also the case that it is not at all clear whether [DMT I] played any role in that death. Whether it was pure coincidence. That's just how it is this with these medications. There is no effective drug without side-effects. And side-effects can include, in the worst-case scenario, death. I think it is something you just have to live with.

proceeding with other DMTs	[pwMS 34]
	But there is something else I must say, I will never, and I really mean it, badmouth any therapy, for it will work well for some other people. […] And that is actually great that we have a few treatments to try out and there will be the one you decide to stick with and there will be one that can help. [pwMS 15]
Discontinuing a DMT\Decision-making process\No benefit from DMT	It really was the case that the medication itself created more stress for me [doing the injections and the lipodystrophy as a side-effect] than the illness itself. And then, as I said, came the point in 2012 that I said, this can't be the way. [pwMS 2]
Discontinuing a DMT\Decision-making process\Effort in the mode of administration	And in 2012, I said that the stress has such a hold that I can't see the good in it, and then I stopped. [pwMS 2]
Discontinuing a DMT\Decision-making process\Adverse effects	And I no longer accepted it. I am now 36, then I was 30, so hey, I'm not an 80-year-old yet, I don't have to move this way. I want to keep on enjoying life, just as it is. [pwMS 4]
Discontinuing a DMT\Decision-making process\Having benign MS	So, I took it from 2001 until 2011 […]. I actually had just one relapse, so that Prof XY said in 2011, I was here again for a clinical re-evaluation, because there were still colleagues who said, no, you don't have MS. And he recommended then that I stop taking [DMT B]. He said […] that benign MS exists as a concept and that in fact the power or the strength of [DMT B] wasn't so great as to be able to create such an excellent disease course. [pwMS 32]
Discontinuing a DMT\Decision-making process\Transition from RRMS to SPMS	I am not willing to embark on the alternatives detailed to me for the secondary progressive course of disease which are currently available on the market, because for the most part they are derivatives of chemotherapies. And that is a little too full-on for me. So, rather lead a healthy life, live well, take things slowly. [pwMS 26]
	The next one would be [DMT K]. But I […] did my own reading on that, communicated with some people who had already experienced [DMT K], or who had information about [DMT K] and for now I've decided against it. […] I think, I'm just going to call it a day for now. I've tried several therapies, […] but such a powerful treatment, such an impactful therapy as [DMT K], I'll give it a wide berth for now. [pwMS 17]
Discontinuing a DMT\Coping with the	It is hard to say which would have been better, or good, stopping all together or continuing with different medication. [pwMS 29]

decision to discontinue treatment with a DMT\State of uncertainty	Yes, it is definitely strange to begin with. Is it the right decision or isn’t it? But as I mentioned, I did dedicate a lot of thinking time to it and no longer having to inject is a real relief. […] In that respect I felt so good, being out and about, and I know the psychological aspect plays an enormous role and I’ve done a lot of research on this and sought advice. [pwMS 2]
Discontinuing a DMT\Coping with the decision to discontinue treatment with a DMT\Improvement of well-being	But there is something I must say, that I feel such a sense of freedom now, and more confident. [pwMS 26]
	I feel a sense of freedom. [pwMS 4]
Discontinuing a DMT\Coping with the decision to discontinue treatment with a DMT\Defending the decision	I know from a friend of mine who is the same age as me, also has MS, a similar course […] I know, you can’t generalise, but although he did everything possible, tried out all possible medications, it got steadily worse right up to his death. Bearing that in mind, I really can’t say that I regret my decision about 15 years ago to stop taking any medication. It’s true, of course, that I can’t stand, that I can’t walk. But my head is still working well. And I am still alive. [pwMS 29]
	I tried several medication therapies, gave it my best shot, didn’t resist them. [pwMS 17]
Discontinuing a DMT\Coping with the decision to discontinue treatment with a DMT\Tolerating impairment	And now I haven’t taken any further medication for three years. […] I want to keep on enjoying life, just as it is. Sure, there are limitations and regarding things through a particular lens, not being blind to, but rather focussing on, why the body is doing this or that now, what could be causing it? Is it caused by the MS or does it have another source? You’ve always got to weight that up carefully, because doctors, in my experience, are very quick to lay everything at the door of MS once that’s the diagnosis. But not everything has to do with MS. That’s my experience at least. You have to learn to listen in deeply to your body, get to know yourself. [pwMS 4]
Discontinuing a DMT\Coping with the decision to discontinue treatment with a DMT\Using other treatment approaches	And if I do then have to deal with more severe relapses, then I’d prefer to rely on a course of cortisone […] Thank God I don’t have any major side-effects with cortisone […] and thus for me it is the best alternative for the future, for dealing with the illness. [pwMS 17]
	So I do other things […] I smoke joints, […] I take sulphur, […] I drink lots of ginger tea, ginger […] turmeric, I go more for the natural remedies, that nature provides. [pw MS 4]
MS without starting a	At the beginning of the 90s, there was no information going. That’s when I thought, okay, seeing as there isn’t anything,

DMT\Decision-making process\Little or no choice of DMTs	then it's not as if I'm carelessly missing a trick, there's nothing for me to do. [pwMS 3]
MS without starting a DMT\Decision-making process\Critical attitude towards conventional medicine and DMTs	Then it turned out over the next few years, there was always one relapse. A single relapse and usually connected to times of intense emotion. And then, of course, at some time or other came the suggestion: "You should think about it", as they put it. And my reply was, I've been thinking about it for years, and I don't want to. Yes, that was my mantra back then: "No, I don't want to." [pwMS 31]
	To begin with I didn't want to start on any drug therapy. I was really clear about that and that's why it was only later on, when the relapses were happening more and more often that I thought it over and thought okay, I probably won't manage it without medication. [pwMS 46]
MS without starting a DMT\Decision-making process\Absence of a recommendation by a physician for a DMT	"Don't give your body any substances, or it will get curious about them. So, try to avoid them as long as you can", and that was how I lived. […] I personally believed at the start that if I were to give my body something, then it would get used to it, and that might reduce the effectiveness of other mechanisms which are also important, and thus I told myself, no. As long as I am not restricted, then I won't take anything. [pwMS 40]
	So, when it comes to these drug therapies, no one ever told me I should take something […]. And I had a neurologist, […] he said himself that he adhered to things that are statistically proven. But if I were to take a different path, that would be fine, too. But it was more at that level; he didn't contradict me. […] And with no one insisting upon it, I stopped […] seeking medication and I simply did nothing. [pwMS 42]
	I should follow a healthy lifestyle. Shouldn't smoke, and should do sport, and I shouldn't eat too much pork. That was the long and the short of my doctor's advice, rather than assigning me medication. [pwMS 18]
MS without starting a DMT\Decision-making process\Benign course of MS	Because it had been only a brief exceptional event, a relapse, that I had back then, and it cleared up pretty much within four to six weeks, and no residual damage. That was the very start of the story. Yes, so I was ill for four weeks and then… Yes, it was simply suppressed then, the MS. And for the first three years I basically didn't take any medication because there was nothing wrong with me. I was healthy, as far as I was concerned. And the neurologist I had then, he also said: "You can take this, or not, as you like." And at that time, when I was diagnosed, there were only two therapies. […] And it was like that: "You can take it or not, either way. We don't know whether it helps." Yes, and when you're 18, 19, or 20, you think "I'm not interested!" And get on with living. [pwMS 23]
MS without starting a	So, no one said to me, there is this and there is this and which would you like, or similar. I was lucky. I believe I was

DMT\Coping with the decision not to start treatment with a DMT\Feeling comfortable with the decision	lucky. It was a stroke of luck for my life that no one forced me to take anything. [pwMS 42]
MS without starting a DMT\Coping with the decision not to start treatment with a DMT\Using other treatment approaches	What I found really great at that time, 2011, 2012, there was also a study, […] it was about incense. […] MRIs with contrast dye was part of that, but then I had half a lesion too few to be able to join the study, in one of the monitoring months. And so, well, that was that. […] At any rate, I actually decided about a year later to buy the stuff myself […] I did that, for two or three whole years, also hoping that it might take the edge off the depressions, but unfortunately that didn't work out. [pwMS 39]
	So, I tried out various things […] for example: […] meditation. You don't simply sit there listening to music and struggling with the thoughts that come into your head, but rather we visualise in our mind what the life we want to live looks like. For our brain cannot differentiate between what is imagined and what is actually taking place and if we give that a positive turn, then we send into our body all the good things it needs to be healthy […] feeling good rather than feeling the fearfulness of a victim's state. [pwMS 2]
MS without starting a DMT\Coping with the decision not to start treatment with a DMT\Tolerating impairment	Sometimes minor relapses occurred, slight distortions in sensation, but nothing that was really of major significance, and it was only over the course of two or three years that the regularity increased a bit. Or was it that I just paid more attention to it or they were more actively perceptible when I saw them on MRI images, where it was always: "oh, here are a couple of bright circles, those were relapses." And then I'd say, "I hadn't noticed, but if you say so, there must have been." [pwMS 40]
MS without starting a DMT\Coping with the decision not to start treatment with a DMT\Starting a DMT	At the beginning, I didn't want to start any medication therapy. I was really clear on that and that's why it was only later on, when the relapses were happening with increasing regularity that I thought to myself, okay, probably you are not going to manage this without medication. [pwMS 46]
